# Associations among Organochlorine Pesticides, *Methanobacteriales*, and Obesity in Korean Women

**DOI:** 10.1371/journal.pone.0027773

**Published:** 2011-11-17

**Authors:** Hae-Sook Lee, Je-Chul Lee, In-Kyu Lee, Hyo-Bang Moon, Yoon-Seok Chang, David R. Jacobs, Duk-Hee Lee

**Affiliations:** 1 Department of Public Health Graduate School, Kyungpook National University, Daegu, Korea; 2 Department of Microbiology, Kyungpook National University School of Medicine, Daegu, Korea; 3 Department of Endocrinology, Kyungpook National University School of Medicine, Daegu, Korea; 4 Department of Environmental Marine Sciences, College of Science and Technology, Hanyang University, Ansan, Korea; 5 School of Environmental Science and Engineering, POSTECH, Pohang, Korea; 6 Division of Epidemiology, School of Public Health, University of Minnesota, Minneapolis, Minnesota, United States of America; 7 Department of Nutrition, University of Oslo, Oslo, Norway; 8 Department of Preventative Medicine, School of Medicine, Kyungpook National University, Daegu, Korea; University of Cordoba, Spain

## Abstract

**Background:**

Although *Methanobacteriales* in the gut has recently been linked to obesity, no study has examined the hypothesis that waist circumference, a marker of visceral obesity, are positively associated with *Methanobacteriales* in the general population. Since *Methanobacteriales* increase in a petroleum-contaminated environment to biodegrade petroleum as one way of autopurification, we also hypothesized that high body burden of highly lipophilic petroleum-based chemicals like organochlorine pesticides (OCPs) is associated with higher levels of *Methanobacteriales* in the gut.

**Methodology/Principal Findings:**

Among 83 Korean women who visited a community health service center for a routine health checkup, quantitative real-time PCR (qPCR) based on 16S rDNA was used to quantify *Methanobacteriales* in feces. Nine OCPs were measured in both serum and feces of 16 subjects. *Methanobacteriales* were detected in 32.5% (27/83 women). Both BMI and waist circumference among women with *Methanobacteriales* were significantly higher than in women without *Methanobacteriales* (P = 0.04 and P = 0.01, respectively). Also, *Methanobacteriales* levels in feces were positively associated with BMI and waist circumference (r = +0.23 and P = 0.03 for both). Furthermore, there were significant correlations between feces *Methanobacteriales* levels and serum concentrations of most OCPs, including with cis-nonachlor (r = +0.53, P<0.05), oxychlordane (r = +0.46, P<0.1), and trans-nonachlor (r = +0.43, P<0.1). Despite high correlations of serum and feces concentrations of most OCPs, feces OCP concentrations were not clearly associated with feces *Methanobacteriales* levels.

**Conclusion/Significance:**

In this cross-sectional study, the levels of *Methanobacteriales* in the human gut were associated with higher body weight and waist circumference. In addition, serum OCP concentrations were positively correlated with levels of *Methanobacteriales*. There may be a meaningful link among body burden of OCP, *Methanobacteriales* in the gut, and obesity in the general population.

## Introduction

Methanogens are microbes that produce methane gas from various substrates such as H_2_ and CO_2_, acetate, and methylamine. They were identified as belonging to the domain archaea in the 1970s [1]. Pathogenicity, such as tissue invasion and toxin release commonly observed with classic pathogens, has not yet been described for methanogens [2]. Although causal pathways are not clear, significant associations have been observed between methanogens and periodontitis, colon cancer, or diverticulosis [2].

Methanogens were recently linked to obesity [3]. Many dietary components that are resistant to initial digestion in the small intestine are subsequently fermented by the microbial community of the large intestine, producing short-chain fatty acids (SCFAs) that are absorbed across the colonic mucosa [4]. These SCFAs have been estimated to provide 10% of daily caloric intake [4]. In experimental mice, the presence of methanogens in the colon promotes calorie harvest and adiposis through improved efficiency of polysaccharide fermentation by the *Bacteroidetes* and the *Firmicutes,* the primary bacterial fermenters in the gut [3]. Fermentation of polysaccharides generates SCFAs, principally acetate, propionate, and butyrate, as well as other organic acids and gases like H_2_ and CO_2_ [5]. Accumulation of H_2_ inhibits bacterial NADH dehydrogenases, thereby reducing the yield of ATP. Studies in man-made bioreactors have shown that removal of H_2_ by methanogens could promote obesity by improving fermentation efficiency of dietary polysaccharides [3].

Despite the clear experimental evidence [3], however, human studies on the associations between methanogens and obesity have shown inconsistent results [6], [7], [8], [9]. One small-scale human study with 9 study subjects also observed an association between methanogens and extreme obesity [7]. However, in the other studies, methanogens were increased in anorectic patients or lean subjects [6], [8], [9]. To our knowledge, no study has explored associations between gut methanogens and waist circumference, a marker of visceral obesity, in the general population. Different from subcutaneous fat underneath the skin, visceral fat surrounding vital organs is strongly linked to various obesity-related chronic diseases [10].

On the other hand, it is unclear which specific environmental factors determine the levels of methanogens in the human gut, although both shared and unique environmental factors have been suggested in a twin study as determinants of the ecology of methanogens in the gut [11]. In the field of environmental microbiology, methanogens have been reported to biodegrade petroleum hydrocarbons in polluted environments [12]. Therefore, this kind of microorganism tends to increase in places with petroleum contamination as one way of autopurification and is artificially used to remove petroleum-based pollutants in environments [13]. Therefore, we hypothesized that increased body burden of petroleum-based man-made chemicals would promote methanogens in human gut.

In relation to this hypothesis, recent epidemiological studies on Persistent Organic Pollutants (POPs), very lipophilic man-made chemicals produced based on petroleum, are highly relevant. For example, environmental exposure to POPs is strongly linked to a variety of chronic diseases and obesity in the general population [14], [15], [16], [17], [18], [19]. POPs are very slowly eliminated from the body via feces, following two major mechanisms of biliary and intestinal excretion [20]. Therefore, persons with a high body burden of POPs may also have higher levels of POPs in their feces. Another direct source of POPs in feces is POPs contained in food. Therefore, it is reasonable that methanogens may increase to biodegrade POPs in the feces, similar to the autopurification commonly observed in petroleum-contaminated environments.

This study was primarily performed to explore if there are relations of *Methanobacteriales,* the predominant methanogenic archaea in the human gut [7], with body mass index or waist circumference in Korean women. Further, we conducted a pilot study to examine the associations between serum or stool concentrations of organochlorine pesticides (OCPs), one subclass of POPs, and *Methanobacteriales* in feces in a subsample of 16 women.

## Methods

### Human subjects and survey methods

We studied 83 Korean women who visited a community health service center for a routine health checkup in Koryung County, Korea. They did not have current gastrointestinal symptoms or a history of chronic gastrointestinal problems. None of them lived in the same household, and none had received any antibiotic or probiotic agents in the 3-month period before the collection of fecal samples. This study was conducted with the approval from the Institutional Review Board at the Kyungpook National University Hospital. Written informed consent was obtained from all study subjects.

Height and body weight were measured using standard methods in light clothes. Body mass index (BMI) was calculated as weight divided by height squared (kg/m^2^). Waist circumference was measured in the supine position midway between the lowest rib and the iliac crest. Blood samples were obtained by venipuncture in the morning after overnight fasting. Participants were also asked to collect the last part of stools when they defecated. After collection, both blood and stool samples were frozen at −70°C until the molecular analyses were conducted. Fasting glucose, triglycerides, and high density lipoprotein (HDL) cholesterol were determined by enzymatic methods using ADVIA 1650 (Bayer Inc., USA).

### Quantification of *Methanobacteriales* in feces

Quantitative real-time PCR (qPCR) was used to quantify *Methanobacteriales*. The genomic DNA was extracted from the frozen stool samples (wet weight 0.2 g) using the QIAamp DNA Stool Kit (Qiagen) according to the manufacturer's instructions. We performed 16S rDNA-targeted qPCR with TaqMan detection kit (Applied Biosystems). The *Methanobacteriales-*specific primers and probe were MBT-NF (5′-TCG CAA GAC TGA AAC TTA AAG GAA-3′), MBT-NR (5′-CGG CGT TGA ATC CAA TTA AAC-3′), and MBT-N-probe (5′-AGC ACC ACA ACG CGT GGA GCC-3′) [7]. Analyses were performed in a total volume of 15 µl containing the following: 7.5 µl of Universal PCR Mater Mix (TaqMan), 1.5 µl of oligonucleotide mixture (4 µM of each primer and 1 µM of FAM-labeled probe), and 1 µl of template DNA. The amplification program consisted of one cycle of 95°C for 10 min and then 40 cycles of 95°C for 15 s and 60°C for 15 s. qPCR was performed in an ABI Prism 7500 cycler (Applied Biosystems). Standard curves were created using serial 10-fold dilution of the standard plasmid DNA corresponding to 2×10^2^ to 2×10^11^ copies/ml. The plasmid DNA standards of target organisms were prepared from the representative 16S rDNA clones, which were amplified from stool samples by specific primers and then cloned into pGEM-T vector (Promega). We calculated the copy number of the 16S rDNA as follows: grams/plasmid molecules  =  (number of base pairs) * (average weight of double stranded DNA)/Avogadro's number and copy numbers/template µl (*q*)  =  (grams/µl)/(grams/plasmid molecules). Finally, copy number per gram of wet stool  =  *q*×*D*×(50 µl)/(0.2 g), where *D* is the dilution factor, 50 µl is the elution volume in genomic DNA extraction, and 0.2 g is the wet weight of stool used for DNA extraction.

### Measurement of OCPs

For analyses of OCPs in serum and feces, we selected 16 out of 83 subjects based on the results of *Methanobacteriales* analyses in feces; 8 from women with *Methanobacteriales* and 8 from women without *Methanobacteriales*. OCPs in serum were analyzed at the laboratory of the School of Environmental Science and Engineering, POSTECH (Pohang, Korea) while OCPs in stool were analyzed at the laboratory of the Department of Environmental Marine Sciences (Ansan, Korea). Both of them used an isotope dilution method with GC-HRMS (gas chromatography-high resolution mass spectrometry). GC-HRMS measurements were performed on a JMS-800D instrument (JEOL, Japan) interfaced with a 6890N gas chromatography (Agilent Technologies, USA).

### Statistical analyses

First, we compared BMI and waist circumference between subjects with and without *Methanobacteriales*. Next, Pearson correlation coefficients were presented between log transformed count of *Methanobacteriales* and BMI or waist circumference. *Methanobacteriales* showed a right-skewed distribution. Also, we presented detection rates of *Methanobacteriales* according to tertiles of waist circumference. Finally, we presented Pearson correlation coefficients between log transformed concentrations of OCPs in serum or feces with log transformed count of *Methanobacteriales*. All data were analyzed using SAS version 9.1.

## Results

*Methanobacteriales* in feces were detected in 27 out of 83 study subjects (32.5%). Among 27 women with *Methanobacteriales*, mean and standard deviation of number of *Methanobacteriales* were 17.6±45.1 (×10^7^). Table 1 shows demographic and clinical characteristics of study subjects depending on the presence of *Methanobacteriales*. BMI or waist circumference of women with *Methanobacteriales* was statistically significantly higher than those of those without them (P = 0.04 and P = 0.01, respectively). There were no significant differences in age, fasting glucose, triglyceride, total cholesterol, and HDL-cholesterol.

**Table 1:** General characteristics by presence of *Methanobacteriales* (n = 83).

	*Methanobacteriales*		
Characteristics	Present (n = 27)	Absent (n = 56)	P value
Mean ± standard deviation			
Age (years)	58.6±7.3	59.3±7.8	0.71
Body mass index (kg/m^2^)	26.4±3.2	24.7±3.5	0.04
Waist circumference (cm)	88.8±7.3	83.6±9.0	0.01
Fasting glucose (mg/dl)	95.6±15.4	92.7±37.7	0.27
Triglycerides (mg/dl)	143.2±65.4	140.7±91.7	0.58
Total cholesterol (mg/dl)	210.6±22.7	204.1±37.5	0.19
HDL-cholesterol (mg/dl)	43.9±9.6	45.6±8.2	0.29
Percentages (%)			
Body mass index ≥25 kg/m^2^	66.7%	46.4%	0.08
Cigarette smoker	0.0%	1.8%	0.48
Alcohol drinker	15.4%	7.4%	0.27

**Figure 1:**
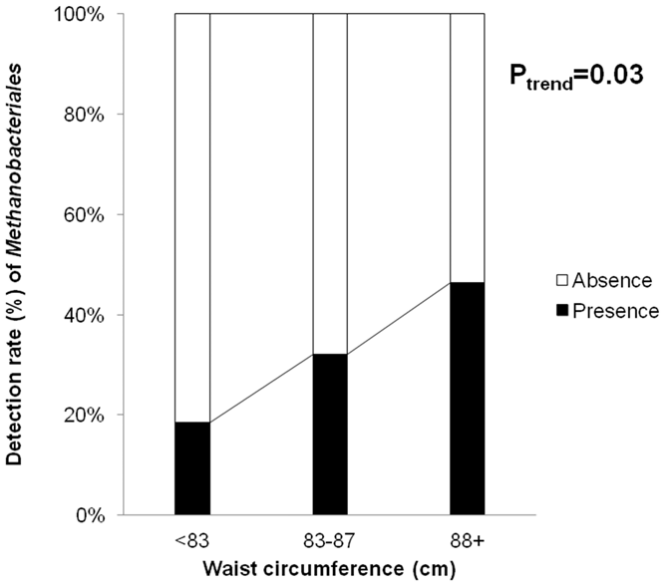
Detection rate of *Methanobacteriales* by tertiles of waist circumference (n = 83).

The number of *Methanobacteriales* had correlation coefficients of +0.23 (P = 0.03) with both BMI and waist circumference, but lower correlations with other characteristics (Table 2). Figure 1 shows the detection rate of *Methanobacteriales* by tertile of waist circumference. Among women with waist circumference <83 cm, *Methanobacteriales* were detected among 18.5% while women with waist circumference 83∼87 cm and ≥ 88 cm showed detection rates of 33.3% and 48.2% (P trend = 0.03).

**Table 2:** Correlation coefficients between levels of *Methanobacteriales* with obesity and obesity-related metabolic variables (n = 83).

Characteristics	Correlation coefficients	P value
Age	−0.08	0.46
Body mass index	+0.23	0.03
Waist circumference	+0.23	0.03
Fasting glucose	+0.07	0.55
Triglycerides	+0.04	0.75
Total cholesterol	+0.09	0.40
HDL-cholesterol	−0.09	0.42

Table 3 shows absolute concentrations of OCPs in serum and feces and their correlations among 16 women selected to balance presence or absence of *methanobacteriales*. As concentrations of OCPs in feces were measured based on dry weight while concentrations of OCPs in serum were measured based on lipid weight or wet weight, direct comparison of concentrations between two specimens was difficult. However, correlation coefficients between serum concentrations of OCPs and those of feces were substantial, in particular with β-hexachlorocyclohexane and OCPs belonging to the Chlordane family. In case of OCPs belonging to the DDT family, both p,p;-DDE and p,p;-DDD showed modest positive associations between serum and feces while p,p'-DDT showed an inverse association between these two specimens.

**Table 3:** Correlation coefficients among serum concentrations of organochlorine pesticides (OCPs), stool concentrations of OCPs, and levels of *Methanobacteriales* (n = 16††).

	Mean ±standard deviation		Correlation coefficients		
Characteristics	OCPs in serum(ng/g lipid)	OCPs in feces‡(pg/g) ‡	OCPs in serum vs. OCPs in feces	OCPs in serum vs. *Methanobacteriales*	OCPs in feces vs. *Methanobacteriales*
*p,p*'*-*DDE	114±54.3	1461±676	+0.42	+0.33	−0.02
*p,p*'*-*DDD	2.1±1.1	235±75.1	+0.24	+0.40	−0.44*
*p,p*'*-*DDT	10.6±5.9	204±116	−0.48*	+0.41	−0.42
Oxychlordane	4.1±1.6	49.0±36.4	+0.62^**^	+0.46*	+0.19
*trans-*Nonachlor	6.7±2.9	101±51.2	+0.51^**^	+0.43*	+0.15
*cis-*Nonachlor	1.2±0.6	29.0±14.6	+0.49*	+0.53^**^	−0.06
Heptachlor epoxide†	7.1±3.2	-	-	+0.27	-
β-Hexachlorocyclohexane	35.5±32.1	2176±1647	+0.89^***^	+0.33	+0.23
Hexachlorobenzene	6.7±3.4	2316±2928	+0.19	−0.07	−0.13

*:P<0.1, **:P<0.05, ***:P<0.01.

† Heptachlor epoxide was measured only in serum.

‡ Concentrations of OCPs in feces were presented based on dry weight.

†† 8 selected at random among the women with no detectable *methanobacteriales*, 8 selected at random among the women with detectable *methanobacteriales*.

**Figure 2:**
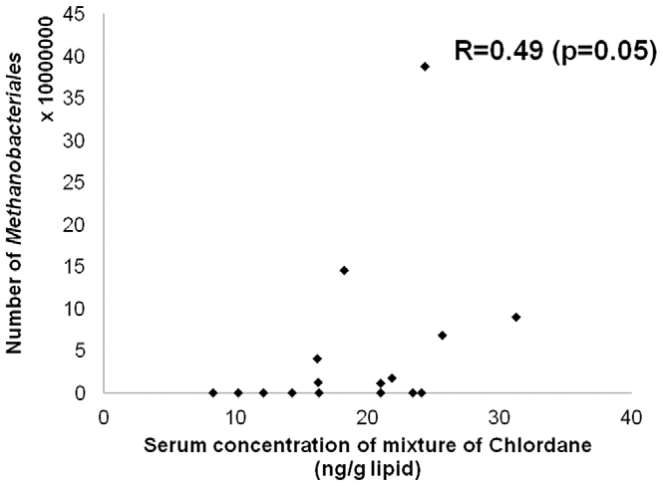
Scatter plot beween serum concentrations of mixture of organochlorine pesticides belonging to Chlordane (oxychlordane, *trans-*Nonachlor, *cis-*Nonachlor, Heptachlor epoxide) and number of *Methanobacteriales* (n = 16). When one subjects with the highest number of *Methanobacteriales* (38.7 × 10^7^) was excluded, the correlation coeffficent was 0.45 (p = 0.09).

Despite the strong correlations between serum and feces, correlation coefficients with levels of *Methanobacteriales* were more strongly observed with serum than with feces concentrations of OCPs (Table 3). Although most OCPs in serum showed positive associations with levels of *Methanobacteriales*, in particular cis-nonachlor (r = +0.53, P<0.05), oxychlordane (r = +0.46, P<0.1), and trans-nonachlor (r = +0.43, P<0.1) showed statistically significant or marginally significant correlations. OCPs belonging to the DDT family also showed correlations coefficients of around + 0.3 to +0.4, although they did not reach statistical significance. Figure 2 shows a scatter plot beween serum concentrations of the mixture of organochlorine pesticides belonging to the Chlordane family (oxychlordane, *trans-*Nonachlor, *cis-*Nonachlor, Heptachlor epoxide) and number of *Methanobacteriales* among 16 women.

## Discussion

In this cross-sectional study, we found that *Methanobacteriales* were positively associated with BMI and waist circumference among Korean women who had mean BMI around 25 kg/m^2^, consistent with the hypothesis that *Methanobacteriales* promote extraction of energy within the human gut. In addition, persons with high *Methanobacteriales* in their feces had higher serum concentrations of OCPs, suggesting that the phenomenon of increased *Methanobacteriales* in an oil-contaminated environment can also be observed in the human gut ecosystem because OCPs are typical highly lipophilic man-made petroleum-based chemicals.

Even though there was a clear trend between waist circumference and the detection rates of *Methanobacteriales,* the correlation coefficient of +0.23 between the number of *Methanobacteriales* and waist circumference can be interpreted as a weak association. However, considering the predominant role of many life style-related factors contributing to obesity, such as excess energy intake and lack of exercise, the strength of association observed in the current study cannot be dismissed as weak.

An experimental study reported that methanogens could promote obesity by improving fermentation efficiency of dietary polysaccharides [3], but, with the exception of one small scale study [7], previous human studies did not support the experimental evidence [6], [8], [9]. In one previous human study with 6 study subjects by Zhang et al, *Methanobacteriales* was detected in all 3 obese persons with mean BMI of 48.3 kg/m^2^, but not in 3 persons with normal weight with mean BMI of 22.7 kg/m [7]. On the other hand, Armougom et al compared 20 obese subjects with mean BMI of 47.1 kg/m^2^, 9 patients with anorexia nervosa with mean BMI of 12.7 kg/m^2^, and 20 normal control with mean BMI of 20.1 kg/m^2^ and reported no difference in the number of *Methanobrevibacter smithii,* the predominant type of *Methanobacteriales* in humans, between obese and normal weight subjects. However, they showed that counts of *M, smithii* were higher among patients with anorexia nervosa with very low BMI compared with normal weight subjects [6]. In a followup study by the same research team, *Methanobrevibacter smithii* was less frequent and significantly less abundant in obese persons with mean BMI of 43.6 kg/m^2^ than controls with mean BMI of 22.1 kg/m^2^ [8]. The depletion of *Methanobrevibacter smithii* among obese healthy subjects with BMI ≥ 30 kg/m^2^ compared to lean subjects with BMI <25 kg/m^2^ also reported by Schwiertz et al [9].

In contrast to the previous studies, which mostly compared mean levels of methanogens between extremely obese patients and normal weight controls, we showed positive associations using continuous BMI or waist circumference and levels of *Methanobacteriales* using correlation coefficients in the general population. Also, considering the distribution of BMI, our participants were closer to control subjects of the previous studies, rather than the extremely obese patients. Therefore, even though at the first glance the current study seems to be consistent with the results of Zhang et al. [7], but not with others [6], [8], [9], our study design is quite different from that of either of the other studies and is the only study to address the associations of adiposity and *Methanobacteriales* within the range that is relevant to most people. Thus, comparison between the current study and the previous studies is difficult.

The adult intestine contains 10 trillion to 100 trillion microbial cells and is dominated by members of just two divisions of bacteria, the *Bacteroidete*s and the *Firmicutes*. A role for the intestinal microbiota in harvesting energy from food [21] and regulating body fat storage [22] was first proposed in rodents. Germ-free mice colonized by microbiota increase their body fat and develop insulin resistance despite a 30% decrease in food intake. These changes were associated with a dysbiosis in obese mice: an increased representation of the *Firmicutes* phylum and a reduced representation of the *Bacteroidetes* phylum [23]. However, modification of the *Fermicutes-to-Bacteroidetes* ratio is not consistently observed in human [24], [25].

In this study, we focused on methanogenic archaea in the gut, rather than the distribution of various microbiota, because we hypothesized that there was a link among POPs, methanogenic archaea, and obesity. In this study, *Methanobacteriales* were detected in 32.5% of study subjects. On the other hand, *Methanobacteriales* were not detected in control subjects in Zhang et al. 's study [7] while *M. smithii* were detected in 63.3% ∼88.9% in control subjects in other studies [6], [8], [9]. As all of these studies quantify the counts of *Methanobacteriales or M. smithii* using RT-PCR on the basis from 16S rRNA sequencing, we do not have any explanation for why there were the different detection rates among the studies. On the other hand, when the methane breathing test was used as an indirect marker of methanogens in gut, it was estimated that methanogenic archaea were present at high levels in 50–85% of humans [26], [27], [28].

One important finding from the current study is the relation between *Methanobacteriales* in feces and serum concentrations of OCPs. To our best knowledge, there has been no previous study which tested this hypothesis. Based on both findings from environmental microbiology which shows the role of methanogens to clear petroleum-contaminated environment by biodegrading petroleum [11], [12] and recent epidemiological findings on POPs [14], [15], [16], [17], [18], [19], we first hypothesized that body burden of POPs would determine the levels of *Methanobacteriales* in feces. To mark POPs body burden, we measured OCPs in 16 subjects because OCPs were most strongly associated with obesity-related metabolic dysfunctions in previous studies [14], [15], [19]. Among OCPs, chemicals belonging to the Chlordane family showed the strongest associations with the levels of *Methanobacteriale* in feces. Interestingly, the Chlordane family was the one which was most strongly and consistently associated with type 2 diabetes in previous epidemiological studies [15], [17]. If OCPs in the gut are really important in determining the levels of methanogens in the gut, the increased levels of *Methanobrevibacter smithii* among patients with anorexia nervosa [6] may be explained because it is reported that weight loss increases serum concentrations of OCPs [29], [30], [31]. Increased serum concentrations of OCPs due to severe weight loss may lead to increased OCP levels and increased methanogens in the gut because concentrations of POPs in feces were positively associated with serum concentrations of POPs as we observed in the current study.

As markers of OCPs in the gut, we measured OCPs in both serum and feces. As far as we know, there has been no previous human study which compared concentrations of OCPs in between serum and feces. In this study, we observed that OCP levels in these two specimens were highly correlated, but only OCPs levels in serum showed significant positive associations with the levels of *Methanobacteriales* in feces. Intuitively, if OCPs in the gut truly increase the levels of *Methanobacteriales* in feces, OCP levels in feces, rather than OCP levels in serum, should have shown stronger associations. However, the results were different. This could be related to poor validity and reliability of OCP levels in feces. To collect feces samples, the subjects were asked to collect the last part of stools when they defecated. However, we cannot be sure how well OCP levels in the collected samples represent OCP levels in gut. OCP levels in feces can differ substantially depending on the frequency and quality of defecation as well as recent diet of study subjects. Also, OCPs would not be homogenously distributed in the total mass of feces. As we can collect only a small part of feces to measure OCPs, OCP levels in collected feces likely do not represent the OCP levels in the whole feces. On the contrary, serum concentrations of OCPs are considered as a marker of total body burden of OCPs [32]. As OCPs in serum keep being excreted through bile acid and large intestine from body [20], OCP levels in serum may better represent the general pattern of OPCs in gut.

There are several study limitations. First, we did not study other diverse bacteria. However, we had a prior hypothesis about relations the body burden of OCPs, *Methanobacteriales* in feces, and obesity. Second, as only women were included in the current study, we cannot say that the current finding can be applied to men. Third, OCPs were measured only in a subsample of study subjects for cost reasons. Even though we did see statistically meaningful associations, the interpretation should be cautious because the result was based on only 16 study subjects.

In this study, we tested the hypothesis which links the body burden of POPs, *Methanobacteriales*, and obesity. The results are consistent with our hypotheses that body burden of OCPs may determine the levels of *Methanobacteriales* in the human gut, and that this process can finally lead to increased body weight and waist circumference. Cautious interpretation should be made about causality of these associations, given the current cross-sectional study design. Also, the association between body burden of OCPs and *Methanobacteriales* in the human gut needs to be confirmed in future studies with more study subjects. Future studies on the role of POPs in the control of methanogens in human gut may be important to understand a more fundamental role of POPs in developing the change of gut microbiota and obesity.
